# *Space-log*: a novel approach to inferring gene-gene net-works using SPACE model with log penalty

**DOI:** 10.12688/f1000research.26128.1

**Published:** 2020-09-21

**Authors:** Qian (Vicky) Wu, Wei Sun, Li Hsu

**Affiliations:** 1 Clinical Research Division, Fred Hutchinson Cancer Research Center, Seattle, WA, 98109, USA; 2 Public Health Division, Fred Hutchinson Cancer Research Center, Seattle, WA, 98109, USA

**Keywords:** Gene-gene network, gene regulation, penalized regression, log penalty, partial correlation, R package, algorithm

## Abstract

Gene expression data have been used to infer gene-gene networks (GGN) where an edge between two genes implies the conditional dependence of these two genes given all the other genes. Such gene-gene networks are of-ten referred to as gene regulatory networks since it may reveal expression regulation. Most of existing methods for identifying GGN employ penalized regression with *L1 *(lasso), *L2 *(ridge), or elastic net penalty, which spans the range of *L1 *to *L2 *penalty. However, for high dimensional gene expression data, a penalty that spans the range of *L0 *and *L1 *penalty, such as the log penalty, is often needed for variable selection consistency. Thus, we develop a novel method that em-ploys log penalty within the framework of an earlier network identification method space (Sparse PArtial Correlation Estimation), and implement it into a R package *space-log*. We show that the *space-log* is computationally efficient (source code implemented in C), and has good performance comparing with other methods, particularly for networks with hubs. *Space-log* is open source and available at GitHub, https://github.com/wuqian77/SpaceLog

## Introduction

The objective of this paper is to introduce a novel method that constructs gene-gene network (GGN) based on high dimensional gene expression data. Popular methods for GGN include neighborhood selection ^1^, graphical Lasso ^2^, and space (Sparse PArtial Correlation Estimation) ^3^. Neighborhood selection consistently estimates the non-zero entries of the partial correlation matrix, and provide an approximation of the maximum likelihood estimate of partial correlation matrix. Graphical Lasso improves on neighborhood selection by providing a maximum likelihood estimate of the partial correlation matrix. The space method exploits the symmetry of partial correlation matrix to improve the estimation accuracy. It also avoids potential conflicts in neighborhood selection, that is, *Y _i_* is selected as a neighbor of *Y _j_* but *Y _j_* is not selected as a neighbor of *Y _i_*, and one has to make a post-hoc decision for whether *Y _i_* and *Y _j_* are connected. Furthermore, those available methods employ *L*_1_, *L*_2_ or elastic net penalty. However, penalties in the range of *L*_0_ to *L*_1_ is often needed to improve the accuracy of variable selection for high-dimensional gene expression data ^4^. In this paper, we propose a new statistical method to estimate GGN by implementing the log penalty for the space approach, and we refer to our method as space-log.

## Methods

Suppose that we have data on *n* independent individuals and *m* genes. Assume the expression of *m* genes, after appropriate normalization, follow a multivariate Gaussian distribution *N*(0, ∑).

### Neighborhood selection using lasso or log penalty: NS-lasso, NS-log

The neighborhood selection (NS) approach considers each gene separately. Let *Y _i_* be the gene expression value for the *i*th gene and *Y*_− *i*_ = ( *Y*_1_, ..., *Y*_*i*−1_, *Y _i_*_+1_, *Y _m_*) *^T^* . For the NS approach, *Y _i_* is regressed on *Y*_*i*_ by a penalized regression:

$$\hat{\beta }=\text{argmin}\;\{\frac{1}{2}{\left(Y_{i}-Y_{-i}\beta \right)}^{T}(Y_{i}-Y_{-i}\beta )+n\sum_{i\neq j}p(\left|\beta_{i,j}\right|;\omega )\}\;(1)$$

with penalty function p(|β|;ω). We will compare NS-lasso with lasso penalty p(|β|;λ)=λ|β| ^5^ and NS-log with log penalty p(|β|;λ,τ)=λlog⁡(|β|+τ) ^6, 7^. Source codes of NS-lasso and NS-log are available at https://github.com/Sun-lab/penalized_estimation/.

### Joint modeling space using lasso penalty: space-lasso

The joint modeling approach space ^3^ is to estimate GGN, without the need to fit many (m) single gene regression models separately, but directly estimate partial correlation among all the genes. Denote the partial correlation between *Y _i_* and *Y _j_* by *ρ*_*i*, *j*_. If we know the concentration matrix ∑ ^−1^ = (σ *^ij^*) _*m×m*_, then $\rho_{i,j}=-\frac{\sigma^{ij}}{\sqrt{\sigma^{ii}\sigma^{jj}}}.$ So we can easily get that $\rho_{i,j}=\text{sign}(\beta_{i,j})\sqrt{\beta_{ij}\beta_{ji}.}$ Thus, the problem is translated into partial correlation matrix estimation. Specifically ^3^, proposed to minimize a penalized loss function

$$L_{n}(\beta ,\sigma ,Y)=\frac{1}{2}\sum_{i=1}^{m}w_{i}{\left\|Y_{i}-\sum_{j\neq i}\beta_{ij}Y_{j}\right\|}^{2}+\sum_{i\neq j}p(\left|\rho_{ij}\right|;\lambda )\;(2)$$

$$=\frac{1}{2}{\sum_{i=1}^{m}w_{i}\left\|Y_{i}-\sum_{j\neq i}\rho_{i,j}\sqrt{\frac{\sigma^{jj}}{\sigma^{ii}}Y_{j}}\right\|}^{2}+\sum_{i\neq j}p(\left|\rho_{i,j}\right|;\lambda ),\;(3)$$

where *w _i_* ≥ 0 is the weight, e.g., uniform weights *w _i_* = 1 for space-no, residual variance based weights *w _i_* = *σ _jj_* for space-res, and degree based weights *w _i_* = number of genes that { *j* : *ρ _i,j_* ≠ 0, *j* ≠ *i*} for space-df. In 3, *p*(| *ρ*|; *λ*) = *λ*| *ρ*| and we call it as space-lasso.

### New algorithm space-log: joint modeling space using log penalty

Inspired by 6 and 7, we extended the space approach with log penalty as space-log *p*(| *ρ*|; *λ*, *τ*) = *λ*log(| *ρ*| + τ) and used the active shooting algorithm ^3^ to update the coefficient estimates iteratively in space-log ( *Extended data*). We determined the tuning parameters by using extended BIC (extBIC) ^8^.

Denote the target loss function as

$$\begin{array}{l} f(\rho ;\sigma )=\frac{1}{2}\sum_{i=1}^{m}w_{i}{\left\|Y_{i}-\sum_{j\neq i}\rho_{i,j}\sqrt{\frac{\sigma^{jj}}{\sigma^{ii}}Y_{j}}\right\|}^{2}+\sum_{i\neq j}p(\left|\rho_{i,j}\right|;\tau ,\lambda )\\ \;=\frac{1}{2}\sum_{i=1}^{m}w_{i}{\left\|Y_{i}-\sum_{j\neq i}\rho_{i,j}\sqrt{\frac{\sigma^{jj}}{\sigma^{ii}}Y_{j}}\right\|}^{2}+\lambda \sum_{i\neq j}\log(\left|\rho_{i,j}\right|+\tau )\;(4) \end{array}$$

The goal is to estimate $\hat{\rho }$ = argmin *_ρ_* *f*( *ρ*) for a given *λ* and *τ*. We implement the penalized estimation using space and Log penalties by Local Linear Approximation (LLA) ^9^.

$$p(\left|\rho_{i,j}\right|;\lambda ,\tau )\approx p(\left|{\hat{\rho }}_{i,j}^{\left(k\right)}\right|;\lambda ,\tau )+p^{\prime }(\left|{\hat{\rho }}_{i,j}^{\left(k\right)}\right|;\lambda ,\tau )(\left|\rho_{i,j}\right|-\left|{\hat{\rho }}_{i,j}^{\left(k\right)}\right|)\;(5)$$

Where $\left|{\hat{\rho }}_{i,j}^{\left(k\right)}\right|$ is the estimate of regression coefficient *ρ _i,j_* at the *k*-th iteration. After applying LLA for the penalty part, we can minimize loss function at the ( *k* + 1)-th step, while solving for *ρ _i,j_* by

$$\begin{array}{l} f^{\left(k+1\right)}(\rho_{ij})=\frac{1}{2}\sum_{i=1}^{m}w_{i}{\left\|Y_{i}-\rho_{i,j}\sqrt{\frac{\sigma^{jj}}{\sigma^{ii}}}Y_{j}-\sum_{l\neq i;l\neq j}{\hat{\rho }}_{i,l}^{\left(k\right)}\sqrt{\frac{{\hat{\sigma }}_{\left(k\right)}^{ll}}{{\hat{\sigma }}_{\left(k\right)}^{ii}}}Y_{l}\right\|}^{2}\\ \;+\sum_{i\neq j}p^{\prime }(\left|{\hat{\rho }}_{i,j}^{\left(k\right)}\right|;\lambda ,\tau )\left|\rho_{i,j}\right|\;(6) \end{array}$$

By letting $\partial \;f^{\left(k+1\right)}(\rho_{i,j})/\partial \;\rho_{i,j}=0,$ we can find the solution for *ρ*_*i*, *j*_ as follows:


$${\hat{\rho }}_{i,j}^{\left(k+1\right)}=\{\begin{array}{ll} 0 & \text{if}\left|z_{j}^{\left(k\right)}\right|\leq v_{j}^{-1}p^{\prime }(\left|{\hat{\rho }}_{i,j}^{\left(k\right)}\right|;\lambda ,\tau )\\ sgn({\hat{\rho }}_{i,j}^{\left(k\right)})[\left|z_{j}^{\left(k\right)}\right|-v_{j}^{-1}p^{\prime }(\left|{\hat{\rho }}_{i,j}^{\left(k\right)}\right|;\lambda ,\tau )] & \text{if}\left|z_{j}^{\left(k\right)}\right|>v_{j}^{-1}p^{\prime }(\left|{\hat{\rho }}_{i,j}^{\left(k\right)}\right|;\lambda ,\tau ) \end{array}\;(7)$$

where $\;z_{i,j}^{\left(k\right)}=Y_{j}(Y_{i}-\sum_{l\neq i;l\neq j}{\hat{\rho }}_{i,l}^{\left(k\right)}\sqrt{\frac{{\hat{\sigma }}_{\left(k\right)}^{ll}}{{\hat{\sigma }}_{\left(k\right)}^{ii}}}Y_{l})/v_{j},\;v_{j}=Y_{j}^{T}Y_{j},$ and $\;p^{\prime }(\left|{\hat{\rho }}_{i,j}^{\left(k\right)}\right|;\lambda ,\tau )=sgn({\hat{\rho }}_{i,j}^{\left(k\right)})\frac{\lambda }{\left|{\hat{\rho }}_{i,j}^{\left(k\right)}\right|+\tau }.$

### Active-shooting

We adapted the same idea active-shooting algorithm from ^3^ to update the coefficient estimation iteratively in space-log. Without loss of generality, we kept most notation from ^3^ but tailored with space-log. The details are included in the *Extended data*.

## Simulation studies

In this section, we present Monte Carlo simulation to evaluate the performance of the space-log, space-lasso, NS-log, and NS-lasso. Following ^7^, we studied two types of graphs: the traditional random graphs (ER model) where all the genes have the same expected number of neighbors ^10, 11^, and hubs graphs where a few genes may have a large number of neighbors (BA model), and BA model is more frequently observed in gene networks ^12^.

We simulated GGN of *m* genes under both the BA and ER models, respectively. The initial graph had one gene and no edge. In the ( *k*+1)th step, we added *e* edges between a new gene and *e* old genes. Under the BA model, there is a greater probability for the new gene to connect to an existing hub gene that has larger number of edges with the probability $p_{E}=v_{i}^{\left(t\right)}/\sum_{j}v_{j}^{\left(t\right)},$ where $v_{j}^{\left(t\right)}$ number of edges connected with the *i*th gene at the *t*th step. For the ER model, each edge of any gene pair ( *G _i_*, *G _j_*) was added randomly in the GGN with probability *pE* independent from all other edges. After constructing the bone of GGN, we simulated gene expression based on multivariate Gaussian. Without loss of generality, we simulated data sets with *n* = 400 individuals. As shown in Table 1, we considered different number of genes *m* = 100, 200, 300 with various sparsity level determined by *p _E_* = 1 *=m* or 2 *=m* for the ER model and *e* = 1 or *e* = 2 for the BA model.

 We evaluated the performance of the methods by the following metrics: number of false positives (FP), false negatives (FN), FP+FN, F1 score, FDR, true positive rate (power). Note that there are three different weights used in joint modeling setting ( space-log, space-lasso): (1) uniform weights; (2) residual variance based weights; and (3) degree freedom based weights. The corresponding methods are referred to as sp_no, sp_res, and sp_df with/without log respectively.

**Figure 1.**
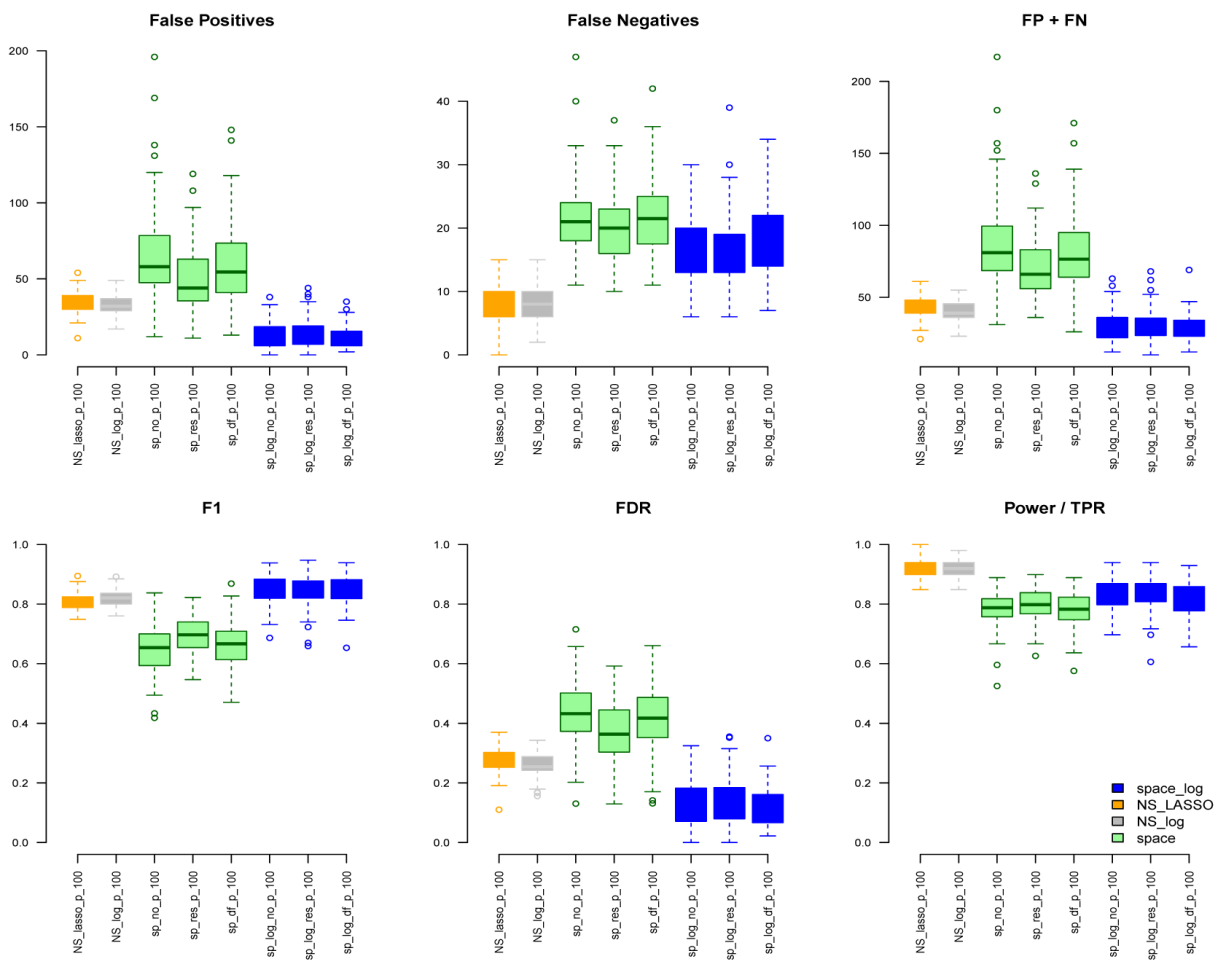
BA with 100 genes and e=1.

**Figure 2.**
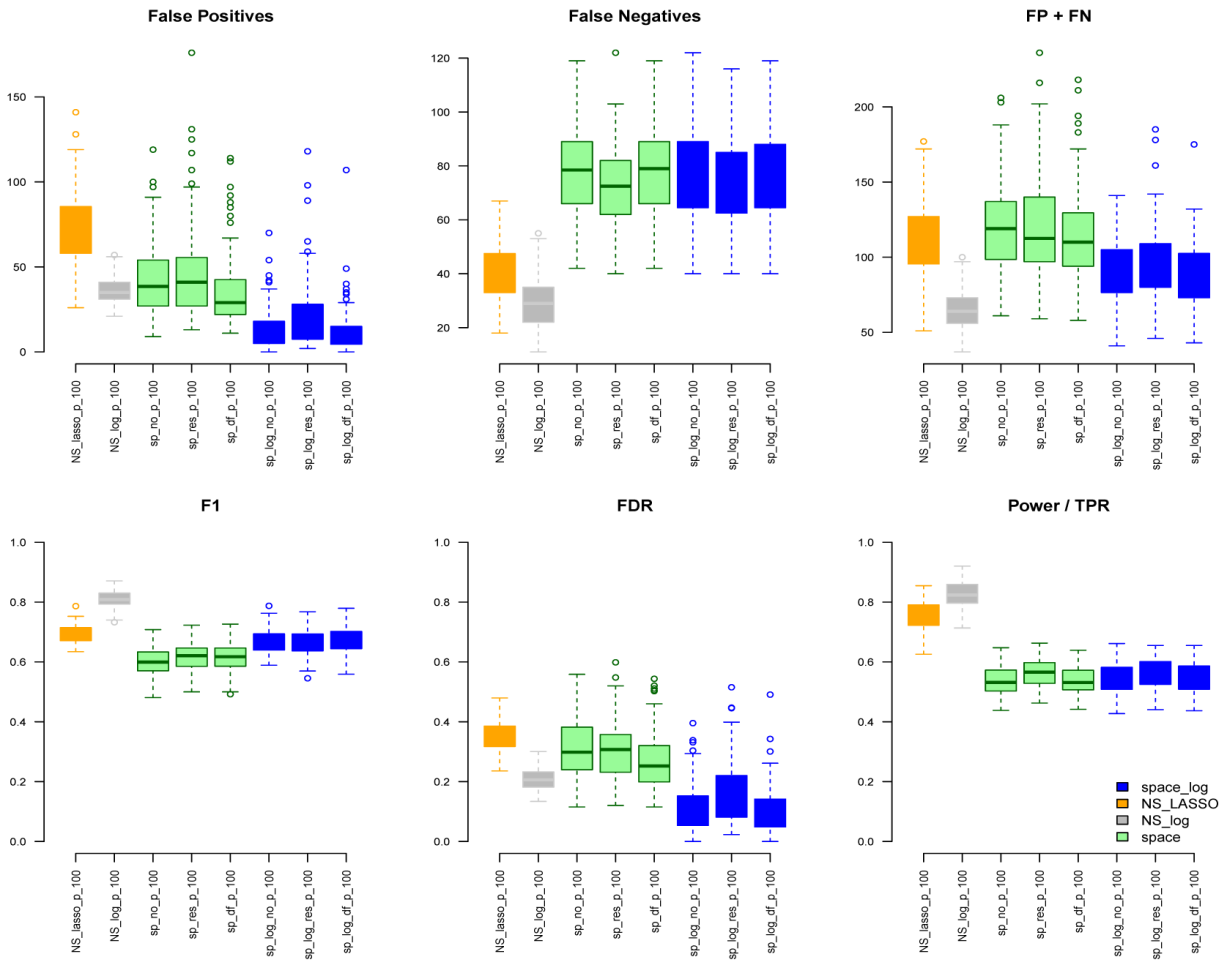
ER with 100 genes and e=1.

Under the BA model with m=100 and e=1 ( Figure 1), we can see that space-log has smallest Errors (FP+FN), smallest FDR, and highest F1 score than other approaches, indicating that space-log controls overall false positive and false negative rates well. Under the ER model ( Figure 2) with m=100 and e=1, space-log is slightly better than space, and NS-log shows lower Errors and higher F1 score than other approaches including space-log. Under both models, the log penalty has less false positives but slightly more false negatives compared to lasso penalty. We note that although log penalty performs well for both the ER and BA models, space-log is particularly powerful in identifying hub networks (such as BA models).

In the *Extended data*, Figures S3 and S4 show the results under the BA model for m=100,200,300 with low number of connections (e=1) and high number of connections (e=2), respectively. Figures S5 and S6 show the results under the ER model with low and high numbers of connections, respectively. Comparing with Figure 1, a similar pattern was noted with the increase of number of genes (m increases from 100, 200, to 300). In BA with low connections (Figure S3), space-log showed smallest FP+FN error and largest F1 score, which outperform all other methods. In BA with high connections (Figure S4), NS-log showed smallest FP+FN error and largest F1 score. For ER model with low and high connections, NS-log outperforms other methods in terms of FP+FN and F1 scores. It’s in line with our understanding that space-log is powerful at identifying hubs network, and NS-log is powerful at dealing with complex network with high number of gene-gene interactions and random networks. 

**Figure 3.**
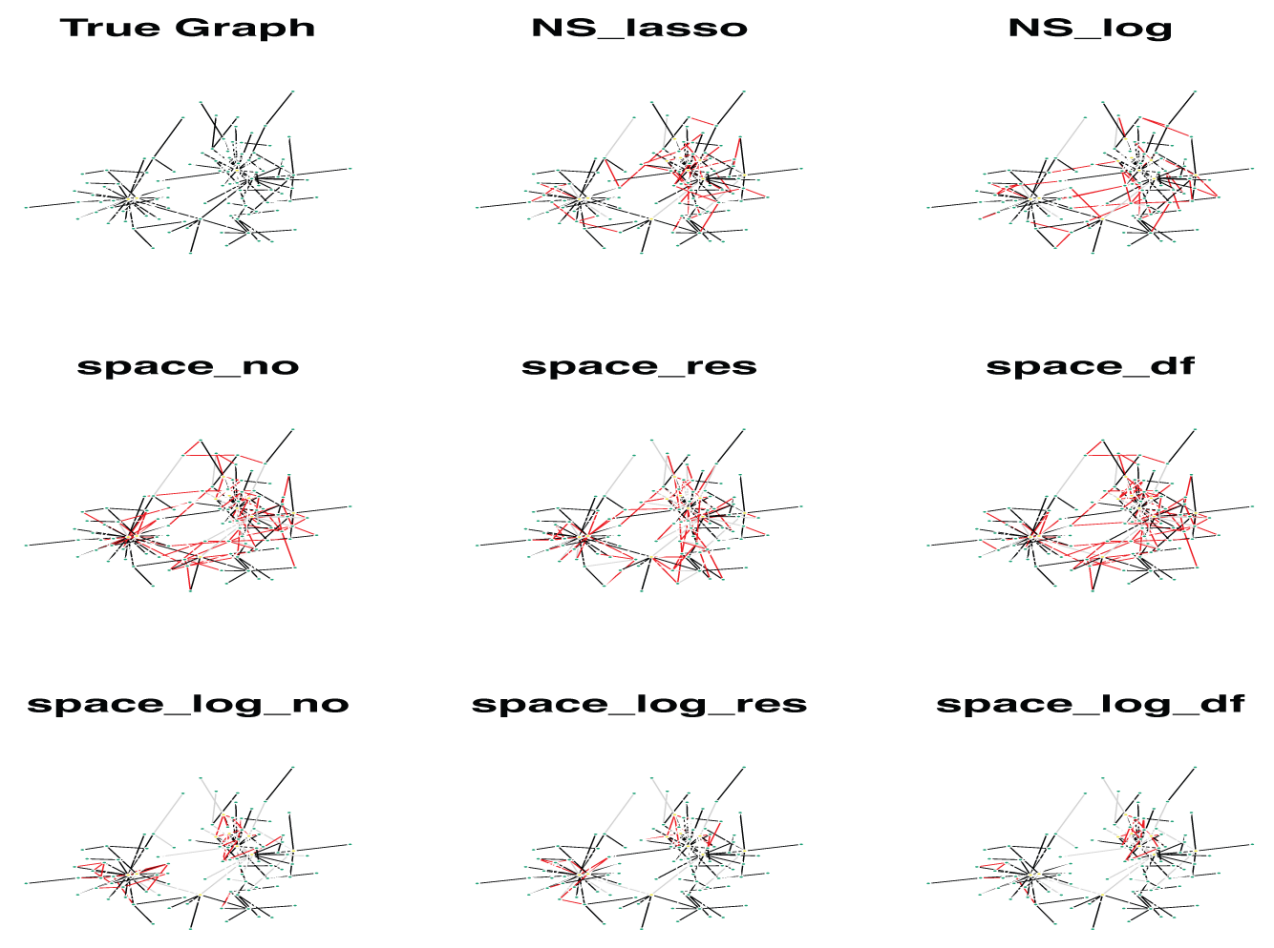
A simulated graph for the BA model with 100 genes (e=1) and multiple hubs. A total of 400 subjects were generated. The GGN was estimated by 8 different approaches. Black is true edges, red is false positive edges, and grey is false negative edges.

We showed a simulated graph for the BA model with 400 subjects, 100 genes and each gene has only 1 connection ( Figure 3). The GGN was estimated by 8 different approaches. In Figure 3, the true edges were indicated by black color, false positive (FP) edges by red color, and false negative (FN) edges by grey color. It’s clear that space-log identified far fewer false positive edges (red line) comparing with space-lasso and NS approaches, while clearly indicating the hub structures. We observed that the FP edges by two NS approaches were quite randomly identified, and the FP edges by two space approaches were mostly within a hub and not between hubs. In summary, the log penalty generally has better performance than the lasso penalty, and both space-log and NS-log control false positive and false negative rate well. For random networks, i.e., no hub, NS-log performs better than other methods. space-log performs best for hub-like gene networks ( Figure 1 and Figure 3) with higher F1 score and less false positive edges. Identifying hub networks is generally considered of great interest in the GGN analysis, because a few of hubs connecting with a large proportion of genes, and those hub genes are thought to be master regulators and play a critical role in a biological system ^13^.

**Table 1.** Simulation settings.

*m*	*n*	*p _E_* (ER)	*e* (BA)
400	100	1/100, 2/100	1,2
400	200	1/200, 2/200	1,2
400	300	1/300, 2/300	1,2

**Figure 4.**
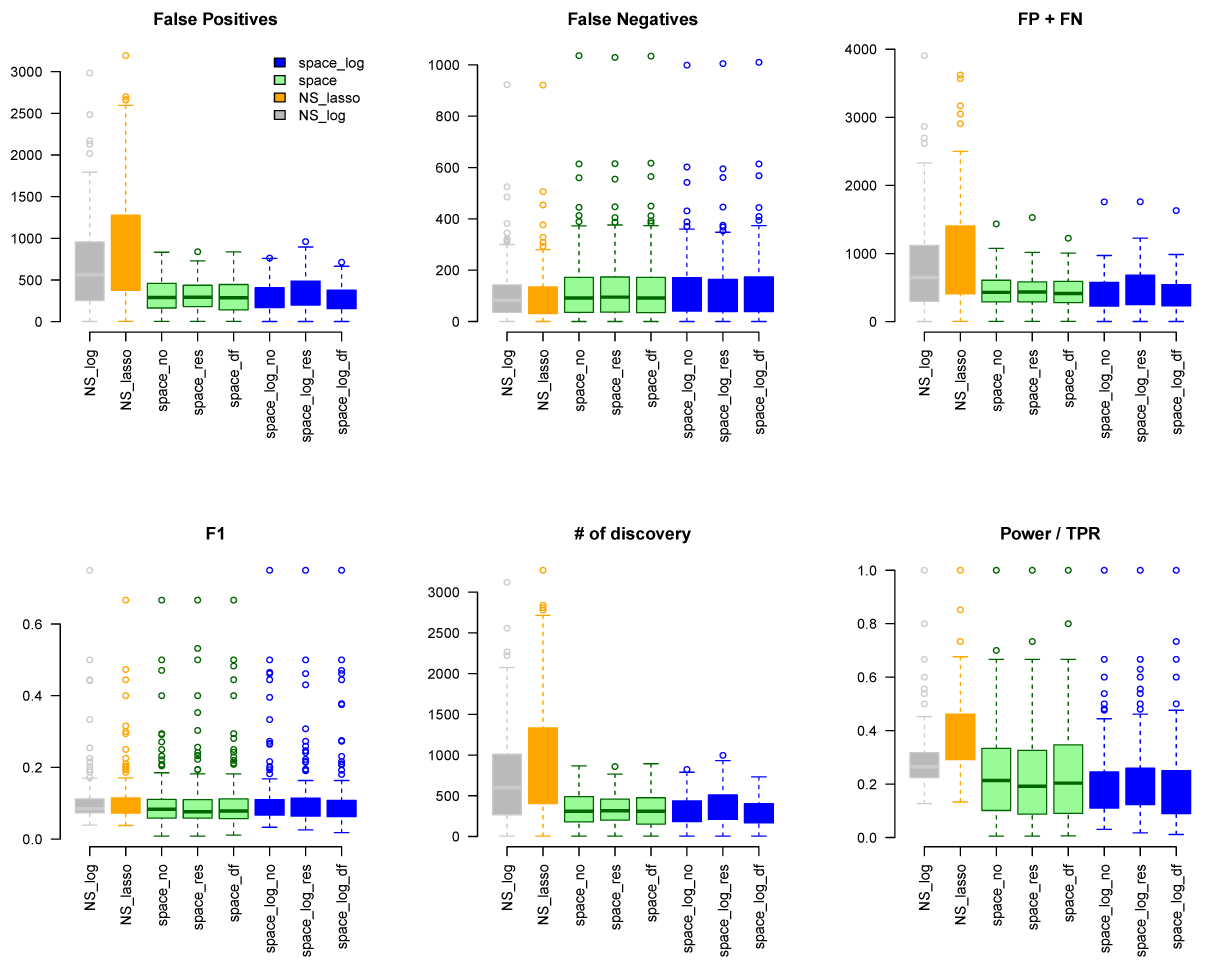
TCGA data analysis with ALL 189 Gene Sets.

**Figure 5.**
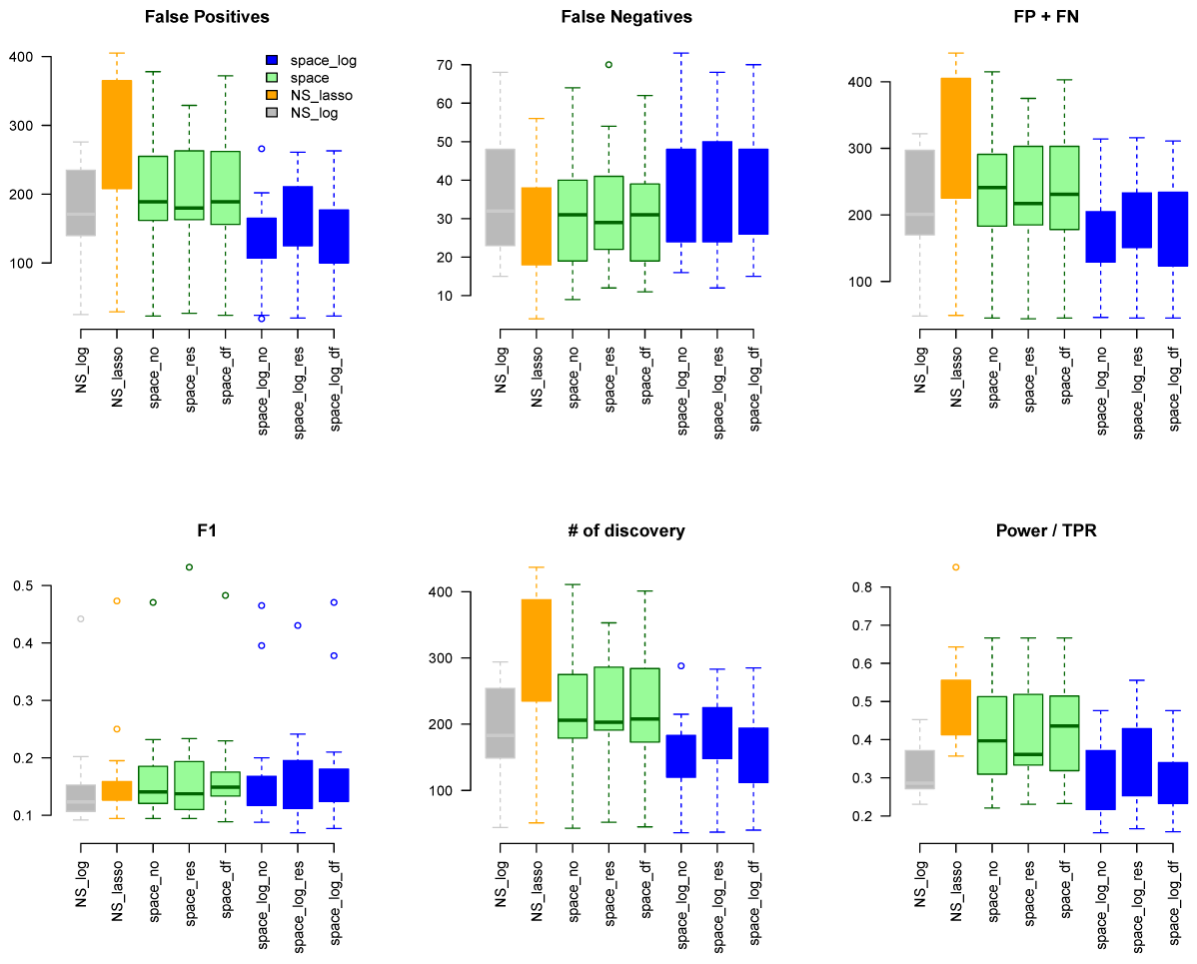
TCGA data analysis with BA hub-type Gene Sets.

**Figure 6.**
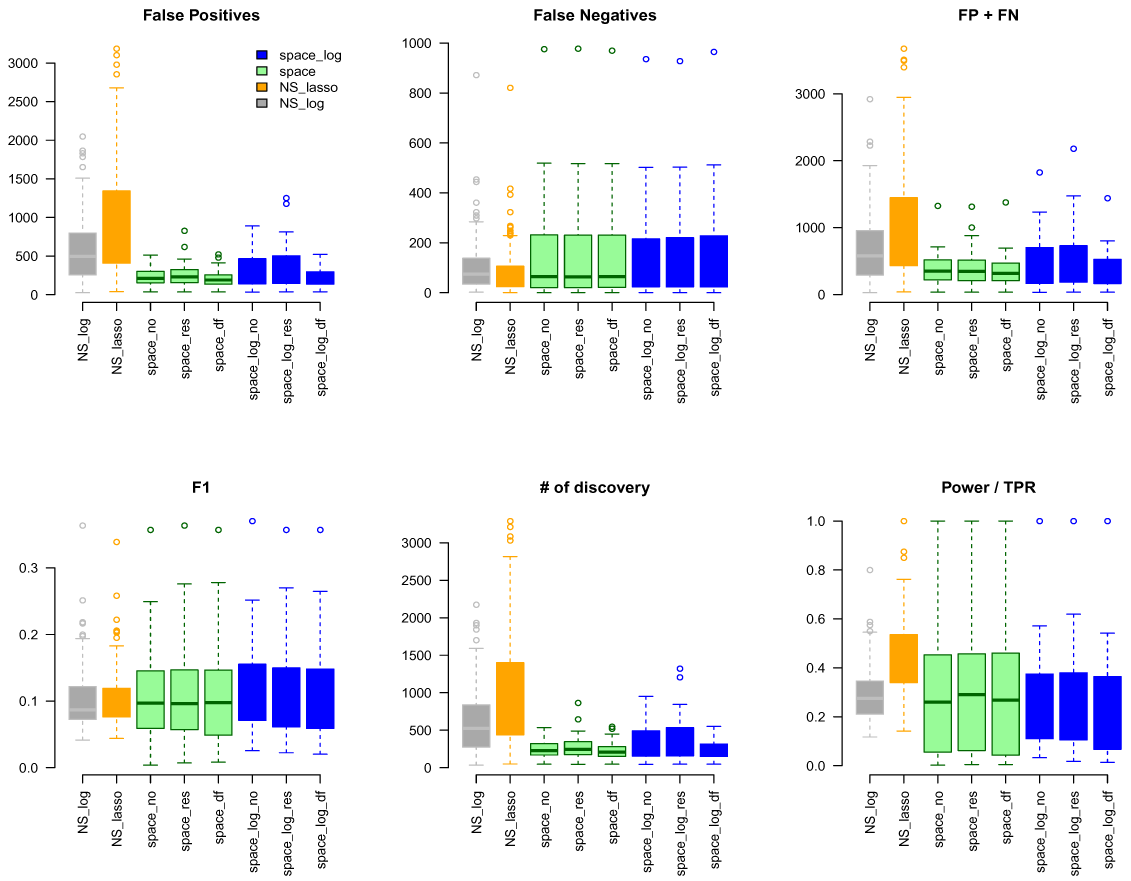
GTEx data analysis with ALL 189 Gene Sets.

**Figure 7.**
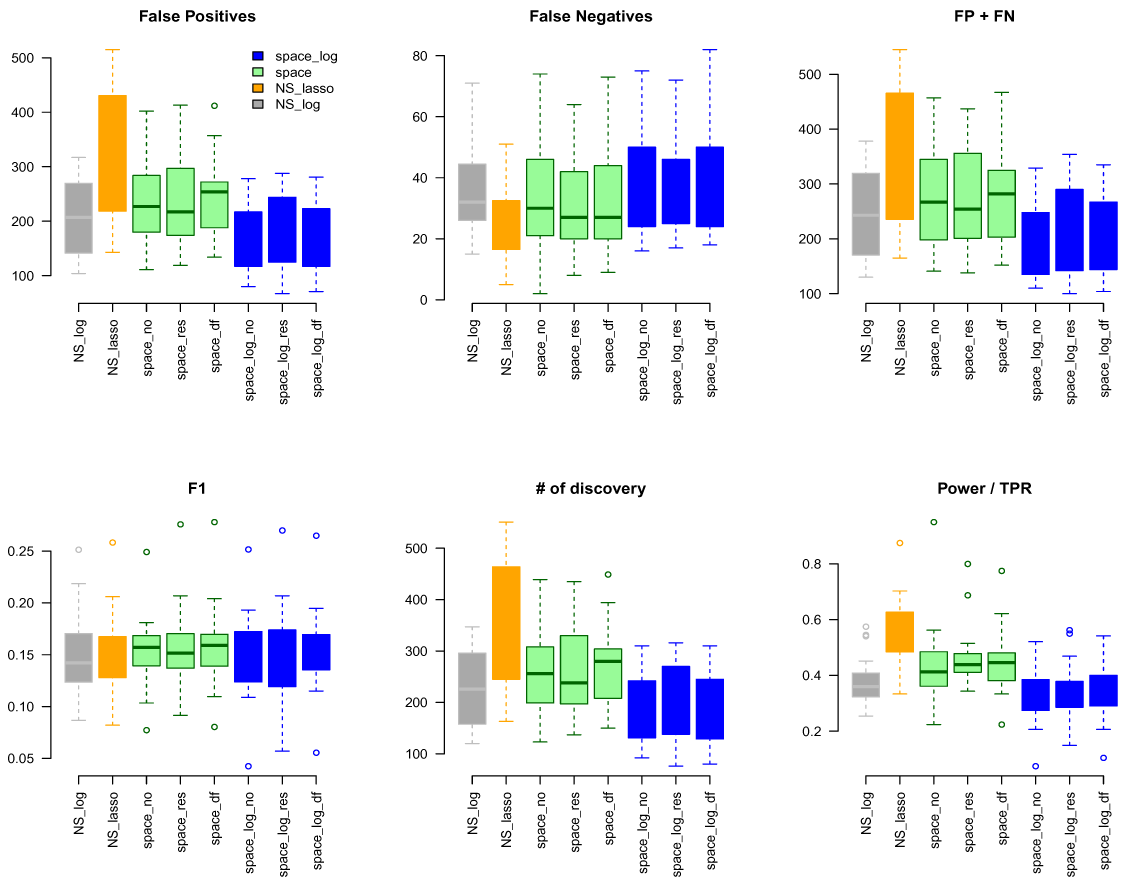
GTEx data analysis with BA hub-type Gene Sets.

## Application to GTEx and TCGA data

### TCGA data

We applied both proposed space-log and existing methods ( space-lasso, NS-log, and NS-lasso) to identify GGN using RNA-seq data from tumor tissue of 550 TCGA (The Cancer Genome Atlas) Colon Adenocarcinoma (TCGA-COAD) cancer patients ^14^. The preprocessing steps of RNA-seq data included: (1) transforming the expression of each gene by log(total read count) = *logTReC* (2) removing the confounding effects by taking residuals of logTReC from a linear regression with the following covariates: 75% of logTReC per sample (which captures read depth), plate, institution, age, and six PCs from the corresponding germline genotype data. After removing genes with low expression across most samples, we had 18,238 genes and 450 samples.

We considered gene sets C6 curated oncogenic pathways by MSigDB from the Broad Institute and inferred the GGN within each gene set. There were 189 gene pathways with a total of 8,737 unique genes for which TCGA have expression data. The sizes of gene sets ranged from 9 to 338 genes. Since we don’t know the true GGN, we downloaded the common pathway version 10 from www.pathwaycommons.org to provide a partial "gold standard". The observed GGN by different methods were compared with the known edges from common pathway and calculated FP, FN, FP+FN, number of total discovery, F1 score, and true positive rate ( *Extended* data *:* Figure S10). The NS-based approach with both LASSO and log penalty discovered much more edges than space-based approach and space-log had fewer false positive (fewer FN+FP too) than space-lasso. There is almost no difference on number of false negative between different methods, as well as F1 score ( Figure 4). Furthermore, in order to show the performance of these methods on the hub networks, we identified 17 pathways with hub-like genes (each hub gene set has < 50 genes and variance of the number of identified edges for each gene in the gene set > the first quartile of all 189 gene sets) and re-calculated the summary metrics in Figure 5. We noted that space-log approach has smallest Errors and slightly higher F1 than other approaches, which is in line with our finding in simulation that space-log is powerful in identifying hub networks, (such as BA models).

### GTEx data

The Genotype Tissue Expression (GTEx) project ^15^ aims to study tissue-specific gene expression and regulation in normal individuals. In this paper, we used gene expression data (RNA-seq) from blood tissue of 451 patients to identify GGN. We pre-processed gene expression data using the same procedure as for TCGA data. We mapped genes to gene pathways by MSigDB ( https://www.gsea-msigdb.org/gsea/msigdb/index.jsp). A total of 189 gene pathways were represented with a total of 8097 unique genes. The size of gene sets ranged from 8 to 306 genes. 

Again, we applied space-log, space-lasso, NS-log, and NS-lasso approaches to identify GGN. Using the same common pathway file used for the TCGA analysis as gold standard, we calculated FP, FN, FP+FN, # of discovery, F1, TPR ( Figure 6). We obtained very similar results to the TCGA data. The NS-based approach with both LASSO and log penalty discovered much more edges than the space-based approach and space-log has fewer false positive (fewer FN+FP too) than space-lasso. There is almost no difference in the number of false negative between different methods, as well as F1 score. A similar sensitivity analysis was conducted to a subset of hub-type genes ( Figure 7), where 30 pathways were selected to be in the first quartile of the variance of the number of identified genes with < 50. It also showed the space-log approach has smallest Errors (F1 is similar to other approaches).

## Conclusions

In this paper, we proposed a new joint modeling method with log penalty, space-log, to identify gene-gene network. An assumption of the GGN analysis is that most of gene pairs do not directly interact with each other, and there are a few of master genes (hubs) for network that connect with many other genes, which are thought to play a critical role in a biological system ^13, 16^. Both simulation and real data analyses showed that space-log is particularly powerful in identifying hub networks and master genes, which is considered of great interest in gene-gene network analysis. In the *Extended data*, we compared several tuning parameter selection approaches, such as BIC Zou *et al.* ^17^, extBIC ^8, 18^, and oracle ^4^, and showed that extBIC outperforms other methods in simulation. The R package "SpaceLog" on GitHub includes algorithms, simulation, and real data examples: https://github.com/wuqian77/SpaceLog.

## Data availability

### Underlying data

***Simulation data*.** We used barabasi.game function from igraph R package to generate the skeleton of a BA model. 

Source code, simulated data, and plots: https://github.com/wuqian77/SpaceLog/tree/master/Simulation.

***TCGA data.*** The RNA-seq dataset from tumor tissue of 550 TCGA (The Cancer Genome Atlas) colon adenocarcinoma (TCGA-COAD) cancer patients ^14^ can be downloaded from dbGap phs000178.v1.p1.: https://www.cancer.gov/about-nci/organization/ccg/research/structural-genomics/tcga.

Pre-processing and data analysis source code: https://github.com/wuqian77/SpaceLog/tree/ master/Analysis/TCGA. 

***GTex data.*** The RNA-seq dataset of blood tissue from the Genotype Tissue Expression (GTEx) project ^15^ can be downloaded from dbGap Genotype-Tissue Expression Project and the study accession is phs000424.v7.p2.: https://www.gtexportal.org/home/datasets. 

Pre-processing and data analysis source code: https://github.com/wuqian77/SpaceLog/tree/master/Analysis/GTex.

### Extended data

Zenodo: SpaceLog: First release of spacelog, http://doi.org/10.5281/zenodo.4002931 ^19^.

This project contains the following extended data:

- the detailed algorithm for active shooting;
- simulation and figures on comparing methods to choose tuning parameters;
- simulation and figures on comparing different GGN methods under various scenarios.

License: GPL-3

## Software availability

Source code for space-log available from: https://github.com/wuqian77/SpaceLog

Archived source code as at time of publication: http://doi.org/10.5281/zenodo.4002931 ^19^. 

License: GPL-3 

Source code for NS-log and NS-lasso available from: https://github.com/Sun-lab/penalized_estimation

License: GPL-3 

Existing methods space-lasso is available on R CRAN: https://cran.r-project.org/web/packages/space/index.html.
