# Supplementary material for: Space-log: a novel approach to inferring gene-gene net-works using SPACE model with log penalty
Source: F1000Res. 2022 Jan 5;9:1159. Originally published 2020 Sep 21. [Version 2] doi: 10.12688/f1000research.26128.2 (PMC8756298; doi:10.12688/f1000research.26128.2)
Supplement: Peer review history

# Peer review: Reviewer response for version 2

**DOI:** 10.5256/f1000research.58701.r118658

**Published:** 2022-01-20

**Authors:** Jessie Jeng

**Affiliations:** 1 Department of Statistics, North Carolina State University, Raleigh, NC, USA

Revisions are sufficient.

Is the rationale for developing the new method (or application) clearly explained?

Partly

Is the description of the method technically sound?

Yes

Are the conclusions about the method and its performance adequately supported by the findings presented in the article?

Partly

If any results are presented, are all the source data underlying the results available to ensure full reproducibility?

Yes

Are sufficient details provided to allow replication of the method development and its use by others?

Yes

Reviewer Expertise:

NA

I confirm that I have read this submission and believe that I have an appropriate level of expertise to confirm that it is of an acceptable scientific standard.

# Peer review: Reviewer response for version 2

**DOI:** 10.5256/f1000research.58701.r118659

**Published:** 2022-01-12

**Authors:** Yuying Xie, Yuning Hao

**Affiliations:** 1 Department of Computational Mathematics, Science, and Engineering, Michigan State University, East Lansing, MI, USA; 2 Amazon, Seattle, WA, USA

The authors have answered all my concerns. Congratulations!

Is the rationale for developing the new method (or application) clearly explained?

Yes

Is the description of the method technically sound?

Yes

Are the conclusions about the method and its performance adequately supported by the findings presented in the article?

Yes

If any results are presented, are all the source data underlying the results available to ensure full reproducibility?

Yes

Are sufficient details provided to allow replication of the method development and its use by others?

Yes

Reviewer Expertise:

Statistics, Biostatistics, Genetics, Bioinformatics

We confirm that we have read this submission and believe that we have an appropriate level of expertise to confirm that it is of an acceptable scientific standard.

# Peer review: Reviewer response for version 1

**DOI:** 10.5256/f1000research.28834.r86957

**Published:** 2021-06-21

**Authors:** Jessie Jeng

**Affiliations:** 1 Department of Statistics, North Carolina State University, Raleigh, NC, USA

This paper considers the problem of identifying gene regulatory networks based on high-dimensional gene expression data. A new method, space-log, is introduced to perform penalized regression with a log penalty. The new method is compared with several existing methods in simulation and real applications using GTEx and TCGA data. The new method seems to outperform other methods in identifying networks with hubs and master genes.       

 The overall presentation is clear. However, several improvements can be made:

1. I think it would be important to explain why the proposed method has advantages in identifying networks with hubs. I would imagine that a network with hubs tends to have more diverse sparsity levels of association across different genes than a network without hubs. Is the proposed method with a log penalty more effective for such scenarios? Or there are other reasons? 
2. The Introduction is a bit too short. I would suggest adding more biological background and a motivation from a real application point of view.  
3. Some discussions on the computational complexity or a comparison of the computational times of different methods should be helpful.

Is the rationale for developing the new method (or application) clearly explained?

Partly

Is the description of the method technically sound?

Yes

Are the conclusions about the method and its performance adequately supported by the findings presented in the article?

Partly

If any results are presented, are all the source data underlying the results available to ensure full reproducibility?

Yes

Are sufficient details provided to allow replication of the method development and its use by others?

Yes

Reviewer Expertise:

Statistics, Statistical Genomics

I confirm that I have read this submission and believe that I have an appropriate level of expertise to confirm that it is of an acceptable scientific standard, however I have significant reservations, as outlined above.

# Response

**Published:** 2021-12-02

**Authors:** Qian Wu

**Affiliations:** Fred Hutch, USA

1. I think it would be important to explain why the proposed method has advantages in identifying networks with hubs. I would imagine that a network with hubs tends to have more diverse sparsity levels of association across different genes than a network without hubs. Is the proposed method with a log penalty more effective for such scenarios? Or there are other reasons? 

*We totally agree with the reviewer that hub type network has more diverse sparsity (BA type graph has a larger variation (number of edges per gene) than ER type graph) and one additional evidence that log penalty works better for diverse sparsity are the results of an earlier paper https://www.ncbi.nlm.nih.gov/pmc/articles/PMC5628772/ e.g., the results in tables 1 and 2.*

 2. The Introduction is a bit too short. I would suggest adding more biological background and motivation from a real application point of view.  

*Good comment. We added biological background in the introduction section accordingly.*

 3. Some discussions on the computational complexity or a comparison of the computational times of different methods should be helpful.

*We thank the reviewer for pointing out this. We have added the run-time discussion with a table in the online manuscript, and a figure in Supplementary Materials on Github. https://github.com/wuqian77/SpaceLog/blob/master/Document/F1000Research_Journal_Article_Spacelog_Supplementary_Materials.pdf*

# Peer review: Reviewer response for version 1

**DOI:** 10.5256/f1000research.28834.r71864

**Published:** 2020-10-19

**Authors:** Chi Song

**Affiliations:** 1 College of Public Health, Division of Biostatistics, The Ohio State University, Columbus, OH, USA

In this paper, the authors developed “space-log”, a new method to infer gene-gene network (GGN), by incorporating log penalty into the “space” method. The authors compared their method to the original “space” with LASSO penalty and the neighborhood selection (NS) methods, using simulation and real data. In general, the description of the proposed method is clear, and the simulation and real application settings made sense. However, I believe this paper needs to be revised to (1) provide the rationale for choosing the combination of “space” and log penalty, (2) compare with more state-of-the-art methods, and (3) improve the presentation and writing. Here are some specific comments.

1. The authors need to discuss why they decided to combine “space” and log penalty for GGN detection. What is the benefit of using log penalty over other penalty choices, including concave penalties and other nonconcave penalties such as SCAD, MCP, and TLP? The introduction should be extended to include more state-of-the-art methods, such as adaptive LASSO or high-dimensional regression methods with sparse precision matrix estimation.
2. Although briefly mentioned gLasso in the introduction, the authors did not talk about this class of methods in the rest of the paper. I am curious to see how space-log compares to gLasso and its extensions with nonconcave penalties in simulation and real application (see Fan *et al.*, 2009). ^1^
3. Based on my understanding, the major benefit of using nonconcave penalties is to reduce the estimation bias of the correlation coefficients. I suggest the authors include comparisons based on the coefficient estimation. In addition, the authors should provide details about how the precision matrices are simulated. Currently, only the simulation methods for its bone structure are provided.
4. In conclusion/discussion, the authors should provide more insights or heuristics about why the proposed method performed better than other methods. What aspects of the data makes the proposed method favorable? Is there any limitation that the users should pay attention to when applying this method in application?

 

**Minor comments:**

1. The citations in this paper are apparently converted from an author-year citation format. The authors need to make changes to the writing to adapt to numbered citation format.
2. Presentation and writing issues, for example:

- The authors need to decide if a comma is needed between two subscripts in the notation (e.g. \beta_{ij} or \beta_{i,j} on page 3 left column).
- On page 4, left column, third paragraph, the sentence “The initial paragraph …” only applies to BA model, but not ER model.
- Also, it should be “in the (t+1)th step” instead of “in the (k+1)th step”. 
- In the last sentence of this paragraph, it should be “1/m” and “2/m”.
- This is not an exhaustive list of writing issues. I suggest the authors proofread carefully.

Is the rationale for developing the new method (or application) clearly explained?

Partly

Is the description of the method technically sound?

Yes

Are the conclusions about the method and its performance adequately supported by the findings presented in the article?

Partly

If any results are presented, are all the source data underlying the results available to ensure full reproducibility?

Partly

Are sufficient details provided to allow replication of the method development and its use by others?

Yes

Reviewer Expertise:

Biostatistics, Bioinformatics

I confirm that I have read this submission and believe that I have an appropriate level of expertise to confirm that it is of an acceptable scientific standard, however I have significant reservations, as outlined above.

## References

[1] : Network exploration via the adaptive LASSO and SCAD penalties. *The Annals of Applied Statistics* .2009;3(2) : 10.1214/08-AOAS215 521-541 10.1214/08-AOAS215 PMC310578221643444

[2] : Sparse Gaussian Graphical Models.2013; 10.1002/9781118573617.ch5 121-140 10.1002/9781118573617.ch5

# Peer review: Reviewer response for version 1

**DOI:** 10.5256/f1000research.28834.r71868

**Published:** 2020-10-14

**Authors:** Yuying Xie, Yuning Hao

**Affiliations:** 1 Department of Computational Mathematics, Science, and Engineering, Michigan State University, East Lansing, MI, USA; 2 Amazon, Seattle, WA, USA

The paper introduces an extension of the SPACE (Sparse Partial Correlation Estimation) method, which is used to estimate a gene-gene network (GNN). The proposed framework, space-log, relies on the log penalty, which delivers better variable selection performance than LASSO, especially for GGN with hubs. The authors also have created a very efficient R package for the proposed method. The paper is clearly written, and the proposed method showed promising results for real data analyses. I list my questions below:

1. In both TCGA and GTEx analyses, the dimensions m is smaller than the sample size n. Since log penalty can handle high dimension low sample size data, can you also include a real application with m larger than n? 
2. The numerical results are all based on the tuned model using extended BIC, which is one-shot of the result. It is better to also include ROC curves reflecting the whole spectrum of the results for a range of tuning parameters.

**Minor comments:**

1. In formula (3), the ‘square root’ should not include Yj. 
2. In t page 3 section ‘Joint modeling space using lasso penalty: space-lasso’, it is better to include the relationship between \beta_{ij} and \simga_{ij} before making the conclusion that \rho_{i,j} = sign(\beta_{i,j}) \sqrt{\beta{ij}\beta_{ji}}.
3. For the log penalty, please also cite ‘Enhancing sparsity by reweighted L1 minimization’.

Is the rationale for developing the new method (or application) clearly explained?

Yes

Is the description of the method technically sound?

Yes

Are the conclusions about the method and its performance adequately supported by the findings presented in the article?

Yes

If any results are presented, are all the source data underlying the results available to ensure full reproducibility?

Yes

Are sufficient details provided to allow replication of the method development and its use by others?

Yes

Reviewer Expertise:

Statistics, Biostatistics, Genetics, Bioinformatics

We confirm that we have read this submission and believe that we have an appropriate level of expertise to confirm that it is of an acceptable scientific standard, however we have significant reservations, as outlined above.

# Response

**Published:** 2021-12-08

**Authors:** Qian Wu

**Affiliations:** Fred Hutch, USA

1. In both TCGA and GTEx analyses, the dimensions m is smaller than the sample size n. Since log penalty can handle high dimension low sample size data, can you also include a real application with m larger than n? 

We thank the reviewer for pointing out this limitation.  We “create” some larger network by combining some gene sets with overlapping genes (m > n).  An updated “larger size network” graph is added in supplementary.

https://github.com/wuqian77/SpaceLog/blob/master/Document/F1000Research_Journal_Article_Spacelog_Supplementary_Materials.pdf

1. The numerical results are all based on the tuned model using extended BIC, which is one-shot of the result. It is better to also include ROC curves reflecting the whole spectrum of the results for a range of tuning parameters.

Good comment. Cross-validation (CV) approach is commonly used to choose the tuning parameter but time-consuming, and recent literature (Wang et al., 2009) showed BIC-type approach has better performance than CV.  Thus, we tried different tuning parameters and used grid search to generate an “Oracle” result (based on maximize F1 score or minimize FDR) in supplementary (section S2.3).  It showed extBIC performs better than BIC and its performance is close to Oracle for space approach. https://github.com/wuqian77/SpaceLog/blob/master/Document/F1000Research_Journal_Article_Spacelog_Supplementary_Materials.pdf

**Minor comments:**

1. In formula (3), the ‘square root’ should not include Yj. 
2. In t page 3 section ‘Joint modeling space using lasso penalty: space-lasso’, it is better to include the relationship between \beta_{ij} and \simga_{ij} before making the conclusion that \rho_{i,j} = sign(\beta_{i,j}) \sqrt{\beta{ij}\beta_{ji}}.
3. For the log penalty, please also cite ‘Enhancing sparsity by reweighted L1 minimization’.

We thank the reviewer for pointing out this oversight.  We have updated the manuscript accordingly.
